# Associated Medial Meniscal Injury with ACL Reconstruction Results in Poorer Strength and Jump Tests Outcomes: A 6-Month Analysis of 504 Patients from the MERIScience Cohort

**DOI:** 10.3390/jcm13237251

**Published:** 2024-11-28

**Authors:** Clément Cazemajou, Thibault Marty-Diloy, Nicolas Graveleau, Pierre Laboudie, Nicolas Bouguennec

**Affiliations:** 1 Clinique du Sport de Bordeaux-Mérignac, 33700 Mérignac, France; thibault.marty-diloy@orange.fr (T.M.-D.); nicolasgraveleau@mac.com (N.G.); pierre.laboudie@gmail.com (P.L.); nbouguennec@gmail.com (N.B.); 2 Centre Hospitalier de La Rochelle, 17000 La Rochelle, France; 3 Centre Hospitalo-Universitaire de Poitiers, 86000 Poitiers, France

**Keywords:** anterior cruciate ligament, meniscal injury, meniscal repair, meniscectomy, 6-month evaluation, composite test

## Abstract

**Background/Objectives**: After anterior cruciate ligament reconstruction (ACLR), a 6-month composite test is recommended during rehabilitation before the return to sport, and the influence of a meniscal tear is not known. The hypothesis was that the location and treatment of meniscus injuries could influence the results of the composite test. **Methods**: A retrospective single-center study was carried out of prospectively collected data involving 504 patients who performed a composite test 6 months after ACLR. Isolated ACLR was compared to ACLR with medial meniscus injuries (MM), lateral meniscus injuries (LM), and bimeniscal injuries (BM) using a composite test including a single-leg squat (SLS), a single-leg landing (SLL), a single hop for distance (SHD), a triple hop for distance (THD) and a side-hop test (Side-HT), isokinetic strength tests, and an assessment of the anterior cruciate ligament—return to sport after injury (ACL-RSI). **Results**: Compared with isolated ACLR, MM injury was associated with a quadricipital deficit at a velocity of 240°/s (14% ± 14% vs. 18% ± 18%, *p* = 0.02), hamstring deficit at 30°/s (14% ± 18% vs. 18% ± 18%, *p* = 0.02) and an increase in the hamstring/quadricipital ratio at 240°/s (68% ± 27% vs. 80% ± 67% *p* = 0.02). Furthermore, ACLR + MM or ML injuries in the operated knee generated an increase in the dynamic valgus frequency detected by the SLS, respectively (40% ± 49% vs. 51% ± 50%, *p* = 0. 05) and (40% ± 49% vs. 54% ± 50%, *p* = 0.02). Meniscal repair and meniscectomies showed no differences. **Conclusions**: These results show that meniscal injuries lead to muscle imbalance for MM injuries and impaired neuromuscular control for MM and LM injuries and suggest that meniscal repairs should be done. Moreover, rehabilitation must be adapted to meniscus injuries.

## 1. Introduction

Injuries of the anterior cruciate ligament (ACL) are associated with meniscus and cartilage damage in 30 to 50% of patients [1,2]. ACL injuries frequently occur in a young, athletic population [3], and the number of ACLRs has recently increased and is expected to continue to rise [4]. Therefore, to obtain reliable and reproducible results, management aims to enable a return to sport at the same level [5] while guaranteeing a low rate of graft rupture and contralateral rupture [6,7].

Various clinical parameters have demonstrated their relevance in assessing a return to unrestricted sport after ACLR: general knee examination, muscle strength single-leg hop test, Lachman rating, and a validated questionnaire [8]; however, isolated, these parameters cannot ensure a safe return to sport [9]. Hence, the consensus group suggested the use of composite tests, including a clinical and psychological assessment, one-foot jump tests, and strength tests for the evaluation of this purpose [10]. As a result, several composite tests have been developed [11,12] and are generally carried out at 6 months after ACLR [13].

The literature shows that these injuries can lead to long-term degenerative osteoarthritis [14]. To reduce this risk, ACL reconstruction (ACLR) combined with meniscal repair whenever possible is recommended rather than meniscectomy [15]. In the short term and medium term, combined ACLR and meniscal injuries have demonstrated a significant impairment of patient-reported outcome measures (PROMS) [16,17], which appeared to be barely affected by meniscal repair or meniscectomy [18]. Consequently, in order to improve the assessment of the return to sport of patients with meniscal injuries as an index of ACLR, some studies have analyzed the results of composite tests according to treatment performed on the meniscal injuries and have not shown any influence of meniscal repairs and meniscectomies on PROMS (self-reported knee-specific instrument and psychological scale), strength tests, and jump tests [19,20]. However, these studies include mainly [19] or exclusively [20] patients who have undergone ACLR using a bone–patellar tendon–bone (BPTB) graft. Furthermore, Redler et al. [21] analyzed 165 patients who underwent ACLR with a hamstring autograft and demonstrated an adverse effect of meniscal repair on hamstring muscle strength. However, all patients who underwent meniscal repair in the study had limited range of motion and weight-bearing during the first 6 weeks post-operation, which may also be one of the contributing factors to these results.

In our large cohort, to adjust rehabilitation protocols for a return to sport, we investigated the relationship between meniscal injuries and composite test results using strength evaluation, jumps tests, and anterior cruciate ligament—return to sport after injury (ACL-RSI). We hypothesized that meniscal injury concomitant to ACLR would lead to lower performance for each parameter of the composite test and that results after meniscal repair would be better than after meniscectomy. The main objective of our study was to investigate whether there is a change in the performance of the composite test when ACLR is associated with a medial meniscus (MM) injury, lateral meniscus (LM) injury, or bimeniscus (BM) injury, in comparison to when ACLR is associated with no meniscal injury. In a second analysis, a comparison was carried out between meniscal repairs and meniscectomies in both the medial and lateral menisci.

## 2. Materials and Methods

### 2.1. Study Design

We studied a retrospective, monocentric study group with prospectively collected data, including patients who underwent ACLR performed by two senior surgeons (NG, NB) at the Clinique du Sport de Bordeaux-Mérignac, using the same technique for ACLR, the same indications for meniscal repair or meniscectomy, and the same protocol of rehabilitation and prospective follow-up. Institutional review board approval was granted for this study (IRB: “CERC-VS-2024-10-1”). Inclusion criteria were: ACLR using a hamstring reconstruction with a four-stand semitendinosus graft (ST4) associated or not with anterolateral ligament reconstruction (ALL) with or without meniscal procedure; patients agreeing to pay for and undergo a composite test 6 months post-operation and no previous history of trauma or surgery on the contralateral knee (non-injured knee). Exclusion criteria were: multiple ligament injuries; incomplete follow-up; absence of socio-demographic, clinical, or radiological data; patient unwilling to pay for the test; patient unwilling to participate; patient with no contact details or history of trauma of surgery on the non-injured knee.

### 2.2. Study Population

All 504 patients underwent surgery between April 2015 and December 2019. All the patients completed a 6-month composite test, and 6 patients were lost during follow-up. The surgical indication was determined after a pre-operative consultation with the completion of a full clinical examination of the knee, a radiological analysis, including AP and sagittal X-rays and MRI (magnetic resonance imaging). When necessary, an anterolateral procedure was pre-operatively planned in accordance with the recommendations of the ALL-study group [22]. Meniscal tear was confirmed, described, and classified according to pre-operative exploration in 3 groups (MM injury, LM injury, and BM injury) and was classified depending on treatment, either by meniscectomy or meniscal repair [23].

### 2.3. Surgical Technique

All procedures were standardized using a hamstring short graft (ST4) [24]. ACLR procedure was performed using a femoral tunnel drill from the inside out, leaving a socket 25 mm deep and a complete tibial tunnel carried out with an outside-in technique. Tibial and femoral tunnels were cut to the size of the graft and fixed with two suspensory buttons: Pullup^®^ and Pullup XL^®^ (SBM, Lourdes, France) [25]. Reconstruction of the anterolateral ligament used a gracilis graft, previously shaped and fixed to the tibia with an ACL Tight Rope^®^ II suspensory button (Arthrex, Naples, FL, USA), and to the femur with a BioComposit FastThreadTM Interference Screw (Arthrex, Naples, FL, USA). The femoral entry point was 1 cm posterior and proximal to the lateral epicondyle, and the tibial one was posterior to the Gerdy tubercle 7 mm below the joint line [26]. When a meniscal injury was diagnosed, the treatment chosen depended on the patient characteristics, the type of meniscal tear, and its location based on international consensus recommendations [27]. Meniscal injuries left in situ corresponded to those affecting only one joint surface (incomplete meniscal injuries), those in the process of healing, or stable vertical injuries to the MM and the posterior horn of the LM. Meniscal tears in Zone 1 (red–red) were repaired as far as possible using an all-inside meniscal repair system Air+ (Stryker, Greenwood Village, CO, USA) [28] using the classic or additional arthroscopic approach. Meniscectomies were performed sparingly using a duckbill clamp and regularized with shaving for Zone 3 (white–white) meniscal injuries, meniscal flaps, and degenerative injuries. The posteromedial compartment was systematically explored to diagnose and describe ramp lesions according to the Thaunat et al. classification [29]. Type 1 was repaired with a minimum of 2 separated sutures performed using a 25° curved hook from QuickPassTM SutureLassoTM (Arthrex, Naples, FL, USA) loaded with a No. 0 absorbable monofilament suture via a posteromedial approach [30].

The same postoperative protocol was applied for all patients: no immobilization or brace, no range-of-motion restriction even if a meniscal repair was performed, and immediate weight-bearing authorization except for radial tears, which required 1 month without weight-bearing.

### 2.4. Outcome Assessment

Follow-up was standardized with clinical evaluation at 1, 3, and 6 months. The 1-month postoperative clinics checked the healing of the wounds, recovery of a complete extension, and a 90° knee flexion. The 3-month clinics aimed to validate the start of re-athletization based on the validation of range-of-motion recovery and strength of quadricipital and hamstring muscles. At 6 months, all patients were invited to complete an individual assessment for a fee of approximately USD 60, based on a composite test including isokinetic evaluation, one-foot tests, and psychological evaluation using the ACL-RSI score [31].

Isokinetic analysis used a Cybex© motorized computerized dynamometer (Cybex Humac Norm 2009 and 2015) [32] to measure quadricep and hamstring muscle strength. Regarding quadricipital evaluation, concentric work was recorded at low velocity (60°/s) to assess strength and at high velocity (240°/s) to assess endurance. Regarding hamstring evaluation, concentric work was recorded at low velocity (60°/s) and high velocity (240°/s), along with low-velocity (30°/s) eccentric work.

One-foot tests included the single-leg squat test (SLS) [33] and the single-leg landing test (SLL) [34], which involves landing on a 35 cm step and then immediately performing another jump. Patients were video-recorded, allowing sports physicians to look for any occurrence of dynamic valgus during movement, which was noted as present or absent. Dynamic valgus was absent if the patella landed in line with the first toe and present if the patella moved inwards and ended up medial to the first toe [35]. One-foot jump tests included the single hop for distance (SHD) test, which measures the maximum distance covered after a stabilized one-leg jump, and the triple hop for distance (THD) test, which is performed and validated under the same conditions after three uninterrupted jumps. The last test performed was the side-hop test (side-HT), which assesses endurance by performing a maximum number of side-hops over 30 s.

For the main analysis, patients were separated into four groups according to the meniscus affected at the time of ACLR: isolated ACLR, which constitutes the reference group, ACLR + MM, ACLR + LM, and ACLR + BM. Meniscal treatments were compared for both medial and lateral injuries.

### 2.5. Statistical Analysis

Data are reported in accordance with STROBE Guidelines [36]. Data were summarized using descriptive statistics, including count and percentages for categorical variables. Continuous variables were described using mean and standard deviation (SD) and categorical variables were presented with total count and percentages. The Chi-squared and Fisher’s exact tests were used to test for differences between categorical variables, and the Kruskal–Wallis test was used for continuous variables.

Statistical significance was set at *p* < 0.05. All analyses were performed using IBM SPSS (Statistical Product and Service Solutions) software for Windows (version 27).

## 3. Results

### 3.1. Patient Characteristics

**Figure 1:**
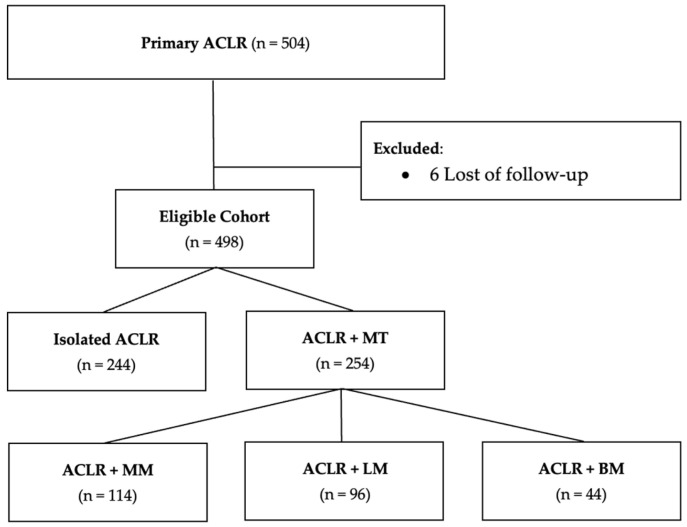
Flowchart of 504 patients describing exclusion criteria and the formation of analysis group. ACLR, Anterior Cruciate Ligament Reconstruction; BM, Bimeniscus; LM, Lateral Meniscus; MM, Medial Meniscus; MT = Meniscal Tear; n, number.

Statistical analysis included 498 patients. Baseline characteristics showed a 27.6 ± 1.9-year-old (14–58) competitive population with a mean pre-operative Tegner Activity Scale of 7.1 ± 0.9 (2–9). There were 254 patients (51.0%) with meniscal tears at the time of ACLR, divided into three groups: isolated MM injury (114 patients, 44.9%), isolated LM tears (96 patients, 37.8%), and BM tears (44 patients, 17.3%) (Figure 1). Among patients with injuries to the MM, 75 patients (65.8%) had a meniscal repair, and 39 patients (34.2%) had a meniscectomy. Among patients with injuries to the LM, 62 patients (64.6%) had a meniscal repair, and 34 patients (35.4%) had a meniscectomy (Table 1).

### 3.2. Effects of Meniscal Injuries on Isokinetic Tests (Table 2)

Patients with injuries of the MM showed:

- A significant quadricipital deficit at a velocity of 240°/s (14% ± 14% vs. 18% ± 18%, *p* = 0.02),
- A hamstring (H) deficit at a velocity of 30°/s (18% ± 18% vs. 14% ± 18%, *p* = 0.02),
- And an imbalance in the hamstring/quadricipital (H/Q) ratio (68% ± 27% vs. 80% ± 67% *p* = 0.02).

Patients with BM injuries showed a significant difference in the H/Q ratio in the operated knee (78% ± 32% vs. 68% ± 27%, *p* = 0.03).

**Table 2:** Results of isokinetic outcomes with meniscal status.

Variable	Isolated ACLR (n = 244)	ACLR + MM (n = 114)	*p* Value	ACLR + LM (n = 96)	*p* Value	ACLR + BM (n = 44)	*p* Value
Quadriceps							
Deficit 60°/s (%)	21 ± 16	22 ± 18	0.53	21 ± 16	0.16	20 ± 18	0.60
Deficit 240°/s (%)	14 ± 14	18 ± 18	0.02	14 ± 14	0.98	18 ± 17	0.09
Hamstrings							
Deficit 30°/s (%)	14 ± 18	18 ± 18	0.02	14 ± 17	0.54	15 ± 17	0.40
Deficit 60°/s (%)	12 ± 14	14 ± 15	0.13	13 ± 16	0.39	11 ± 17	0.90
Deficit 240°/s (%)	08 ± 17	10 ± 17	0.15	16 ± 16	0.16	11 ± 17	0.02
Non-injured H/Q 60°/s ratio (%)	58 ± 42	57 ± 12	0.63	67 ± 97	0.26	59 ± 11	0.95
Operated H/Q 60°/s ratio (%)	64 ± 17	68 ± 29	0.20	63 ± 21	0.52	68 ± 20	0.20
Non-injured H/Q 240°/s ratio (%)	64 ± 21	67 ± 24	0.11	63 ± 23	0.83	71 ± 24	0.04
Operated H/Q 240°/s Ratio (%)	68 ± 27	80 ± 67	0.02	65 ± 27	0.37	78 ± 32	0.03

ACLR, Anterior Cruciate Ligament Reconstruction; BM, Bimeniscus; H, Hamstring; LM, Lateral Meniscus; MM, Medial Meniscus; n, number; Q, Quadricipital. *p* refers to the statistical difference comparing isolated ACLR with ACLR + MM, ACLR + LM and ACLR + BM. Deficit (%) = (uninjured limb − injured limb)/uninjured limb.

### 3.3. Effect of Meniscal Injuries on One-Foot Tests (Table 3)

Analysis of the operated limb of patients with meniscal injuries showed a significantly higher frequency of dynamic knee valgus during SLS for patients with both MM injury (51% ± 50% vs. 40% ± 49%, *p* = 0.05) and LM injury (54% ± 50% vs. 40 ± 49%, *p* = 0.02).

Analysis of the operated limb of patients with BM injuries showed a lower performance at the SHD and THD.

**Table 3:** Results of hop tests with meniscal status.

Variable	Isolated ACLR (n = 244)	ACLR + MM (n = 114)	*p* Value	ACLR + LM (n = 96)	*p* Value	ACLR + BM (n = 44)	*p* Value
Single hop for distance (SHD)							
Non-injured knee (cm)	125 ± 53.9	117 ± 60	0.23	123 ± 58	0.73	92 ± 53.1	<0.001
Operated knee (cm)	111 ± 53.5	103 ± 57	0.22	109 ± 55	0.80	79 ± 49.5	<0.001
Deficit (%)	12 ± 14	12 ± 15	0.75	11 ± 55	0.57	14 ± 15	0.30
Triple hop for distance (THD)							
Non-injured knee (cm)	366 ± 160.9	339.1 ± 174	0.18	359 ± 170	0.73	272 ± 160.1	<0.001
Operated knee (cm)	335 ± 160.2	305.4 ± 165	0.13	315 ± 171	0.34	245 ± 155.4	<0.001
Deficit (%)	09 ± 13	10 ± 11	0.33	10 ±14	0.29	10 ± 14	0.3
Side-hop test (Side-HT)							
Non-injured knee (n)	49 ± 14	46 ± 14	0.14	49 ± 13	0.74	46 ± 15	0.30
Operated knee (n)	42 ± 17	38 ± 17	0.06	43 ± 15	0.57	39 ± 17	0.40
Deficit (%)	16 ± 20	20 ± 23	0.15	13 ± 19	0.29	15.5 ± 41	0.88
Single-leg landing (SLL)							
Non-injured knee (%)	20 ± 40	30 ± 46	0.035	34 ± 48	0.005	21 ± 41	0.82
Operated knee (%)	31 ± 46	39 ± 49	0.11	39 ± 49	0.36	24 ± 43	0.38
Single-leg squat (SLS)							
Non-injured knee (%)	27 ± 44	37 ± 48	0.06	45 ± 50	0.001	19.0 ± 40	0.29
Operated knee (%)	40 ± 49	51 ± 50	0.05	54 ± 50	0.02	45 ± 50	0.49

ACLR, Anterior Cruciate Ligament Reconstruction; BM, Bimeniscus; LM, Lateral Meniscus; MM, Medial Meniscus; cm, centimeter; n, number. Deficit (%) = (uninjured limb − injured limb)/uninjured limb. *p* refers to the statistical difference comparing isolated ACLR with ACLR + MM, ACLR + LM and ACLR + BM.

### 3.4. Effect of Meniscal Injuries on the Non-Injured Limb (Table 2 and Table 3)

We also found significant results for the non-operated knee. Patients with BM injuries reported a significant difference in the H/Q ratio for a velocity of 240°/s (71% ± 24% vs. 64 ± 21%, *p* = 0.04) and poorer performance results at the SHD and the THD.

Furthermore, we noticed a significantly more frequent valgus of the non-injured knee during SLL (30% ± 46% vs. 20% ± 40%, *p* = 0.03) among patients with injuries to the MM, and during both SLL (34% ± 48% vs. 20% ± 40%, *p* = 0.005) and SLS (45% ± 50% vs. 27% ± 44%, *p* = 0.001) among patients with injuries to the LM.

### 3.5. Effects of Meniscal Injuries on ACL-RSI (Table 4 and Table 5)

The ACL-RSI score was not influenced by meniscal injuries and their treatment at the time of ACLR.

**Table 4:** Results of ACL-RSI depending on meniscal tears.

Variable	Isolated ACLR (n = 244)	ACLR + MM (n = 114)	*p* Value	ACLR + LM (n = 96)	*p* Value	ACLR + BM (n = 44)	*p* Value
6 months ALC-RSI (%)	64.5 ± 19.0	63.0 ± 20.0	0.51	66.5 ± 19.7	0.75	66.7 ± 17.8	0.48

ACLR, Anterior Cruciate Ligament Reconstruction; ACL-RSI, Anterior Cruciate Ligament—Return to Sport after Injury; BM, Bimeniscus; LM, Lateral Meniscus; MM, Medial Meniscus; n, number. *p* refers to the statistical difference comparing isolated ACLR with ACLR + MM, ACLR + LM and ACLR + BM.

**Table 5:** Results of ACL-RSI depending on meniscal treatment.

	MM (n = 114)			LM (n = 96)		
Variable	Repair (n = 75)	Meniscectomy (n = 39)	*p* Value	Repair (n = 62)	Meniscectomy (n = 34)	*p* Value
6 months ACL-RSI (%)	64.5 ± 18.8	60.2 ± 22.2	0.27	65.2 ± 19.5	69.0 ± 20.0	0.37

ACL-RSI, Anterior Cruciate Ligament—Return to Sport after Injury; LM, Lateral Meniscus; MM, Medial Meniscus; n, number. *p* refers to the statistical difference comparing meniscal repair to meniscectomy.

### 3.6. Effects of Meniscal Treatment (Table 6 and Table 7)

There were no significant differences for all outcomes regardless of the treatment performed among patients with MM or LM injuries.

**Table 6:** Results of isokinetic outcomes with treatments.

	ACLR + MM (n = 114)			ACLR + LM (n = 96)		
Variable	Repair (n = 75)	Meniscectomy (n = 39)	*p* Value	Repair (n = 62)	Meniscectomy (n = 34)	*p* Value
Quadriceps						
Deficit 60°/s (%)	22 ± 19	24 ± 16	0.55	19 ± 19	17 ± 18	0.56
Deficit 240°/s (%)	17 ± 18	20 ±18	0.44	12 ± 15	14 ± 13	0.44
Hamstrings						
Deficit 30°/s (%)	19 ± 17	18 ± 19	0.84	17 ± 18	14 ± 17	0.84
Deficit 60°/s (%)	14 ± 15	14 ± 15	0.98	12 ± 17	15 ± 14	0.53
Deficit 240°/s (%)	10 ± 17	10 ± 18	0.94	9 ± 16	13 ± 16	0.94
Non-injured H/Q 60°/s ratio (%)	56 ± 12	59 ± 10	0.17	72 ± 22	59 ± 11	0.17
Operated H/Q 60°/s ratio (%)	67 ± 32	68 ± 20	0.88	63 ± 22	63 ± 18	0.88
Non-injured H/Q 240°/s ratio (%)	65 ± 23	70 ± 25	0.93	62 ± 22	65 ± 26	0.25
Operated H/Q 240°/s ratio (%)	80 ± 79	79 ± 34	0.93	63 ± 26	69 ± 29	0.94

ACLR, Anterior Cruciate Ligament Reconstruction; H, Hamstring; MM, Medial Meniscus; LM Lateral Meniscus; n, number; Q, Quadricipital. Deficit (%) = (uninjured limb − injured limb)/uninjured limb. *p* refers to the statistical difference comparing meniscal repair to meniscectomy.

**Table 7:** Results of hop tests with meniscal treatment.

	ACLR + MM (n = 114)			ACLR + LM (n = 96)		
Variable	Repair (n = 75)	Meniscectomy (n = 39)	*p* Value	Repair (n = 62)	Meniscectomy (n = 34)	*p* Value
Single Hop for Distance (SHD)						
Healthy knee (cm)	118 ± 53	118 ± 64	0.79	116 ± 57	134 ± 58	0.79
Operated knee (cm)	105 ± 57	98 ± 59	0.54	104 ± 58	119 ± 50	0.54
Deficit (%)	12 ± 15	13 ± 14	0.83	10 ± 13	13 ± 11	0.83
Triple Hop for distance (THD)						
Healthy knee (cm)	344 ± 171	327 ± 182	0.66	342 ± 168	390 ± 172	0.65
Operated knee (cm)	312 ± 162	290 ± 173	0.54	304 ± 174	336 ± 167	0.54
Deficit (%)	10 ± 11	10 ± 11	0.93	11 ± 15	10 ± 11	0.92
Side-hop test (Side-HT)						
Healthy knee (n)	47.1 ± 13.2	44.0 ± 16.0	0.33	46.9 ± 15.1	53.4 ±12	0.33
Operated knee (n)	39.4 ± 17.1	34.8 ± 17.9	0.24	41.3 ± 15.4	46.9 ± 15.1	0.24
Deficit (%)	19.4 ± 21.5	21.1 ± 25.8	0.73	13.2 ± 20	13.8 ± 17.9	0.73
Single-Leg Landing (SLL)						
Healthy knee (cm)	25 ± 43	42 ± 50	0.07	35 ± 48	33 ± 48	0.07
Operated knee (cm)	33 ± 47	51 ± 50	0.06	42 ± 50	33 ± 48	0.06
Deficit (%)						
Single-Leg Squat (SLS)						
Healthy knee (%)	33 ± 47	43 ± 50	0.31	45 ± 50	44 ± 50	0.31
Operated knee (%)	48 ± 50	57 ± 50	0.39	55 ± 50	53 ± 50	0.39

ACLR, Anterior Cruciate Ligament Reconstruction; MM, Medial Meniscus; LM Lateral Meniscus; n, number. Deficit (%) = (uninjured limb − injured limb)/uninjured limb. *p* refers to the statistical difference comparing meniscal repair to meniscectomy.

## 4. Discussion

This study involved the largest cohort of patients and analyzed the results of a composite test for patients with associated meniscal injuries at the time of ACLR using an ST4 procedure. It showed lower performance in the composite test performed 6 months after ACLR in patients with an MM injury and, to a lesser extent, with an LM injury compared to isolated ACLR, regardless of the type of meniscal treatment. We observed that MM injuries have a negative impact on lower-limb strength flexor muscle, lower extensor limb endurance, endurance imbalance between flexor and extensor, and altered neuromuscular control, whereas LM injuries only alter neuromuscular control.

The population of 498 patients in our study reported a mean pre-operative Tegner at 7.1 and was therefore considered competitive [37]. The proportion of meniscal injuries was comparable to those found in the literature [1]. Meniscal repair was performed on 65.5% of patients with MM injuries and 64.6% of patients with LM injuries, reflecting the ambition of preserving the menisci as much as possible. All patients underwent ACLR using an ST4 hamstring graft, which reported clinical and functional outcomes comparable to other reconstruction techniques [38]. Several studies have analyzed rehabilitation during the first 6 months after ACLR and recommended running composite tests to assess patients’ ability to return to sport, comparable to those used by our team [11]. It is recommended that they include one-foot jump tests assessing vertical and horizontal muscular strength, isokinetic strength tests, and a psychological analysis such ACL-RSI [39]. Therefore, in our study, supervised by a sports physician, all patients completed these tests at 6 months after surgery, prior to the surgeon clinic. Only a few studies have investigated the effect of meniscus damage 6 months after surgery, and this study represents the largest cohort of patients who underwent ACLR using a hamstring-graft standardized method performed by two surgeons.

Isokinetic tests of the operated limb showed that patients with MM injuries had a significantly more important quadricipital deficit during endurance exercise (velocity of 240°/s) than patients with isolated ACLR (14% ± 14% vs. 18% ± 18%, *p* = 0.02). However, at the same velocity, their hamstring performances were comparable. Consequently, we observed a significant imbalance between flexors and extensors, increasing the H/Q ratio by 12% (*p* = 0.02) among patients with MM injuries. To our knowledge, this notion has not previously been demonstrated and must be taken into consideration when adapting the constitution of rehabilitation protocols. The evaluation of patients with MM injuries also showed that the hamstrings of the operated limb had a significant strength deficit at a velocity of 30°/s. Redler et al. also reported that meniscal injuries led to an increase in hamstring deficit in patients undergoing ACLR [21], but their study analyzed meniscal injuries independently of the affected meniscus, using a strength-assessment method other than isokinetic tests, which nevertheless supported our results. LM injuries appear to have no influence on isokinetic tests, whereas BM injuries increase the H/Q ratio at a velocity of 240°/s by 10% (*p* = 0.03) in the operated limb, which may be generated by the effect of MM injuries.

Vertical one-foot jump tests (SLS [33] and SLL [34]) are known to be effective in assessing the quality of the dynamic alignment of the leg by looking for dynamic valgus of the knee, which reflects a loss of neuromuscular control and an increased risk of ACL injury [40]. Therefore, the results of SLS among patients with meniscal injuries at the time of ACLR compared to those without suggest that the presence of meniscal injuries generates a significant loss of neuromuscular control in the operated lower limb at 6 months, regardless of the injured meniscus. This effect has not been demonstrated among patients with bimeniscal injuries, probably due to a too-small population (n = 44).

Moreover, surprisingly, we detected a higher frequency of dynamic knee valgus of the non-injured limb among patients with either an MM or LM injury. Nevertheless, the results of the non-operated knee are difficult to analyze and are multifactorial. We hypothesized that it could be due to a lack of muscle recovery during rehabilitation or to a delay before jumps.

We did not find any similar results in the literature about the consequences of meniscal injuries on the operated knee or on the non-injured knee. These results could lead surgeons and rehabilitation physicians to adapt postoperative rehabilitation in the case of meniscal injury associated with ACLR, especially for jumps and strength recovery.

Horizontal jump tests (SHD, THD, and side-HT) have been widely used to detect asymmetries between the lower limbs [41], and Edwards and al. have shown their predictive effect on return to sport when incorporated into rehabilitation protocols [42]. In our study, no difference was observed between groups except in the group of patients with BM injuries, who showed significantly worse performance in both the non-injured and the operated leg, suggesting that these results were due to physical deconditioning. These non-uniform results between jump tests and isokinetic tests underline the importance of using composite tests, as already reported by Nagai et al. [43].

The ACL-RSI test has demonstrated its clinical relevance in the evaluation of return to sport after ACLR, especially when assessed 6 months after surgery [44], but no cut-off has been yet validated. However, a good return to sport was described for values between 58 and 65%, leaving each team to choose their own threshold for analyzing their patient performance [45]. We could have expected that a meniscal injury at the time of ALCR could have an impact on patient confidence during rehabilitation, but in our study, 6 months after ACLR, meniscal injuries and their treatment, regardless of the affected meniscus, did not appear to have any effect, a fact not previously studied.

Regarding meniscus injury management at the time of an ACLR, the literature has not shown any differences in the evaluation of isokinetic tests and jump tests between patients who have undergone meniscal repair or meniscectomy [19,20]. The study by Moretti and al. compared an isolated ACLR with an ACLR associated with a meniscal repair, and they were the only authors to show that meniscal repair led to a reduction in physical performance, particularly for LM injuries [46]. However, their analysis population remained small, and isokinetic tests were not part of their test battery. Other studies on the same population, with a larger patient volume, showed no adverse effect of meniscal repair on isokinetic tests carried out 6 months after ACLR [47,48]. Our study, in agreement with the literature, shows no difference between meniscectomy and meniscal repair. Therefore, according to the literature and to prevent osteoarthritis after ACLR [14], meniscal repair should be preferred to meniscectomy whenever possible [15,49].

### Limitations

Our study also has some limitations relating to the results of a monocentric patient cohort even if the recruitment area was wide (more than 200 km from the clinic). The injury description did not include some pathological details, which can generate confusion in our subgroup analysis (incidence of cartilage injuries, precise type of meniscal tear). In addition, as BM injuries are less frequent, they constitute a group with a relatively small number of patients, which means that these results must be analyzed with caution. A postoperative rehabilitation protocol with predefined objectives was provided by the surgical center, but the content and frequency of the rehabilitation sessions were left to the discretion of each therapist, who drew up a personalized care plan for the patient. No specific rehabilitation protocol was stipulated, and the quality of rehabilitation could, therefore, be affected, which may have influenced the results of the composite test [50].

The main strength of our study is that it combines a relatively large cohort with a standardized surgical technique performed by only two surgeons. The results were, therefore, reproducible, and the power of the analysis was greater than that of most studies on this subject. This study highlighted the impact that meniscal injuries, especially of the MM, can have on the various components of the composite test and their clinical consequences. It is necessary to consider these results in order to adapt the surgical management of meniscal lesions associated with ACLR and to amend current rehabilitation protocols to overcome the clinical consequences of these lesions. Moreover, meniscal injury continues to appear over time, so a new lesion or a meniscal repair failure may result in a poorer performance on the composite test.

## 5. Conclusions

This study demonstrated that patients with meniscal injuries at the time of ACLR had poorer results on the composite test 6 months after surgery. Our results suggest that injuries of the MM affected both neuromuscular control and muscle performance, resulting in a loss of strength in flexion and an imbalance between the flexor and extensor muscles for endurance exercises, whereas injuries of the LM affected only neuromuscular control with more dynamic valgus.

However, no differences were found between meniscal repair and meniscectomy for both MM and LM injuries, which should lead to a preference for meniscal repair to prevent the risk of osteoarthritis. Consequently, rehabilitation protocols after ACLR should be adapted in the case of meniscus injuries, particularly for MM injuries, in order to restore a correct function during hopping exercises and to improve the muscular balance of the lower limbs.

## Figures and Tables

**Table 1:** Patient characteristics and baseline.

Variable	Values
Age at surgery (years), mean ± SD	27.6 ± 1.9 (14–58)
**Sex, n (%)**	
Male	316 (63.5)
Female	182 (36.5)
Knee right/left, n (%)	267 (53.6)/231 (46.4)
Isolated ACLR, n (%)	244 (49.0)
**ACLR associated with MT, n (%)**	254 (51.0)
MM injury	114 (44.9)
LM injury	96 (37.8)
BM injury	44 (17.3)
**Meniscal treatment, n (%)**	
MM repair	75 (65.8)
MM meniscectomy	39 (34.2)
LM repair	62 (64.6)
MM meniscectomy	34 (35.4)
Anterolateral Ligament Reconstruction, n (%)	153 (30.7)
Pre-injury Tegner Activity Scale, mean ± SD	7.1 ± 0.9 (2–9)

ACLR, Anterior Cruciate Ligament Reconstruction; BM, Bimeniscus LM, Lateral Meniscus; MM, Medial Meniscus; MT, Meniscal Tear; n, number; SD, standard deviation.

## Data Availability

The data that support the findings of this study are available from the corresponding author, Clément Cazemajou.
